# TCM therapies combined with chemotherapy for preventing recurrence and metastasis in postoperative II to IIIA NSCLC: A protocol for a systematic review and meta-analysis

**DOI:** 10.1097/MD.0000000000014724

**Published:** 2019-03-01

**Authors:** Shuntai Chen, Zhenhua Zhang, Xiwen Zhang, Runzhi Qi, Juling Jiang, Xing Zhang, Yupeng Xi, Rui Liu, Qiujun Guo, Honggang Zheng, Baojin Hua

**Affiliations:** a Graduate School, Beijing University of Chinese Medicine; b Department of Oncology, Guang’anmen Hospital, China Academy of Chinese Medical Sciences, Beijing, China.

**Keywords:** complementary and alternative medicine, DFS, lung cancer, OS, RR

## Abstract

**Background:**

Traditional Chinese Medicine (TCM) therapies have been combined with chemotherapy for preventing Recurrence and metastasis in postoperative II to IIIA non-small-cell lung cancer (NSCLC) and the associated better disease-free survival (DFS), but its effects remain elusive. The purpose of this review is to assess the efficacy of TCM therapies as a treatment for postoperative II to IIIA NSCLC.

**Methods and analysis:**

Seventh databases will be searched for relevant studies from inception to the present date. We will include randomized controlled trials assessing TCM therapies combined with chemotherapy for preventing Recurrence and metastasis in postoperative II to IIIA NSCLC. The methodological qualities, including the risk of bias, will be evaluated using the Cochrane risk of bias assessment tool, while confidence in the cumulative evidence will be evaluated using the Grading of Recommendations Assessment, Development and Evaluation (GRADE) approach.

**Ethics and dissemination:**

Ethical approval is not required, as this study is based on the review of published research. This review will be published in a peer-reviewed journal and disseminated both electronically and in print.

**PROSPERO registration number:**

The protocol for this systematic review has been registered on PROSPERO under the number CRD42019116594.

## 1 Introduction

Lung cancer is the most common malignant tumor worldwide, with more than 1.8 million new cases and almost 1.6 million deaths estimated in 2012.^[1]^ More than one-third of all newly diagnosed lung cancers were in China,^[2]^ which was the leading cause of cancer mortality for both men and women in the country.^[3]^ Non-small-cell lung cancer (NSCLC) accounts for 80% to 85% of all cases of lung cancer. About 40% of patients with stages I to IIIA NSCLC, which may be amenable to resection and are potentially curable.^[4,5]^ Current guidelines recommend the use of adjuvant chemotherapy as the care standard for patients with stages II to IIIA NSCLC who have undergone resection.^[6]^ However, despite complete resection and adjuvant chemotherapy, there remains a high risk for developing recurrent diseases with such method. As a result, 40% to 65% of patients with stage II to IIIA NSCLC have experienced recurrence after resection and may ultimately die from disease progression.^[7]^

We aim to assess the impact of TCM therapies on the prevention of Recurrence and metastasis in postoperative II to IIIA NSCLC. TCM therapies are composed of Traditional Chinese Medicine (TCM) decoction and Chinese patent medicine. Both are based on the Chinese Medicine New Medicine Clinical Practice Guideline (Trial Implementation) (published by China Medical Science Press in 2002) and TCM theory of combination of disease and syndrome.

At present, the high rate of recurrence and metastasis of postoperative NSCLC patients are among the primary causes for failures of lung cancer treatments. Besides adjuvant chemotherapy, many Chinese patients also use TCM during or after receiving cancer treatments. Some clinical trials have proved that TCM can be used to prevent recurrence and metastasis after NSCLC resection. Although several studies have been conducted, the effects of TCM therapy in postoperative II to IIIA NSCLC on DFS remain elusive.

## 2 Method

### 2.1 Study registration

**Figure 1:**
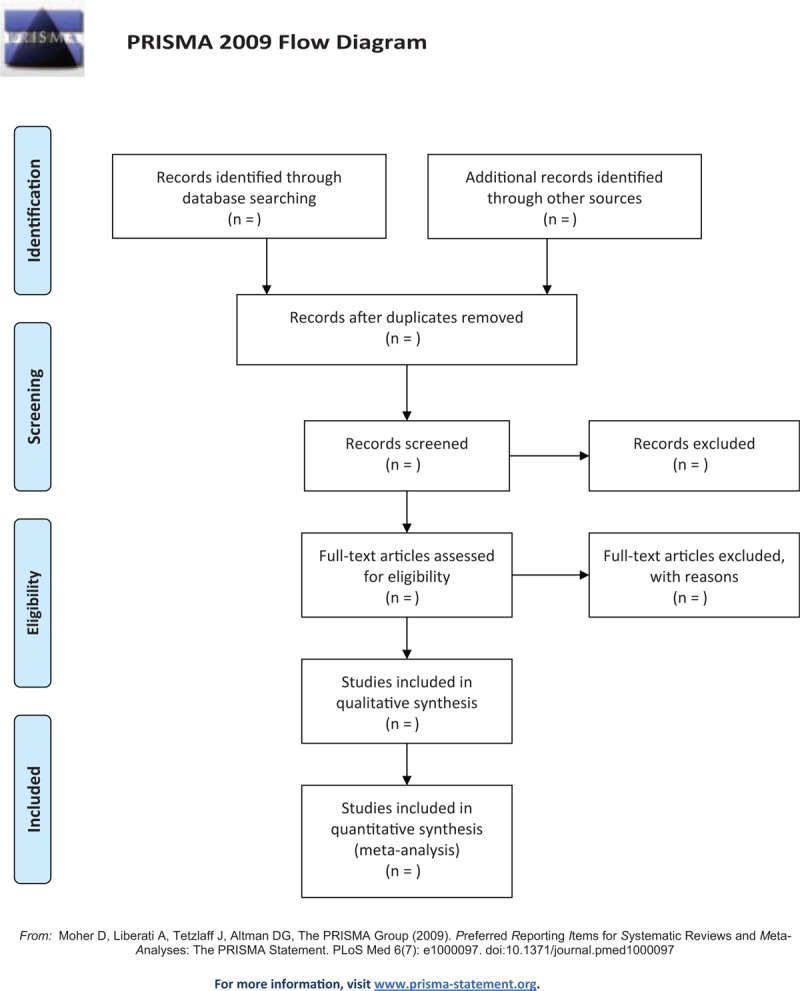
Flow diagram of studies search and selection.

This study will follow the guidelines outlined in the preferred reporting items for systematic reviews and meta-analysis (PRISMA) statement for meta-analyses of healthcare interventions;^[8]^ additionally, the protocol adheres to the PRISMA Protocols (PRISMA-P).^[9]^ The selection process will be summarized according to PRISMA flow diagram (Fig. 1).

The protocol for this systematic review has been registered on PROSPERO under the number CRD42019116594

### 2.2 Types of studies

Randomized controlled trials (RCTs) regarding TCM therapies plus chemotherapy for postoperative II to IIIA NSCLC will be included without restriction language.

### 2.3 Types of participants

Patients were histologically confirmed stage II and IIIA NSCLC after surgical resection, and TNM classification was based on American Joint Committee on Cancer^[10]^ and over 18 years old.

### 2.4 Types of interventions

Intervention: TCM therapy combined with chemotherapy. TCM therapy interventions, including TCM decoction and Chinese patent medicine. We will exclude acupuncture because acupuncture is barely used in preventing Recurrence and metastasis in postoperative II to IIIA NSCLC. Comparator: conventional chemotherapy alone.

### 2.5 Types of outcome measures

#### 2.5.1 Primary outcomes

Disease-free survival (DFS)

Overall survival (OS).

#### 2.5.2 Secondary outcomes

Adverse effects.

Change in symptoms as measured with validated questionnaires.

Quality of life as measured using a validated questionnaire.

## 3 Search methods for the identification of studies

The Cochrane Library, MEDLINE, Embase, Chinese BioMedical Database (CBM), China National Knowledge Infrastructure (CNKI), Chinese VIP Information (VIP), Wangfang Database will be searched regardless of publication date, or language.

## 4 Data collection and analysis

### 4.1 Selection of studies and data extraction

All the included studies will be screened by 2 investigators to extract the following data: last name of the author, publication time, study design, comparator, study period, numbers of outcomes, sex, age, smoking status, locations, histologic diagnosis, TNM stage, BMI, FACT-L4.0 score, and KPS score duration of TCM therapies, timing of TCM therapies, chemotherapy regimens, duration of follow-up, and relevant indicators of bias risk assessment. If above-mentioned information is not able to get, we will contact the corresponding author for detailed data. If there are divided questions or opinions between 2 investigators will be resolved through discussion with the third researcher.

### 4.2 Assessment of risk of bias in included studies

We will use Cochrane Collaboration tool^[11]^ to assess the risk of bias. Each included study will be evaluated respectively by 2 researchers. Random sequence generation, allocation concealment, subjects and researchers blinded, outcome evaluation of blind method, the result data are incomplete and selective report results and other issues are involved and classified as “low,” “high,” or “unclear” based on Cochrane Collaboration tool. If there are divided opinions between 2 researchers in procession, we will resolve inconsistencies through discussion or asking for a help from a senior researcher.

### 4.3 Measures of treatment effect

We will apply relative risk (RR) to represent the enumeration data; measurement data will be represented by mean difference (MD) and 95% confidence interval (95% CI).

### 4.4 Dealing with missing data

Corresponding authors will be connected by E-mail for detailed data if their studies’ information is not available. If no additional message is received, we will conduct data synthesis using available data.

### 4.5 Assessment of quality in included studies

The quality of each selected studies will be evaluated using the Grading of Recommendations Assessment, Development and Evaluation (GRADE) approach by 3 investigators.

### 4.6 Assessment of heterogeneity

Random models will be applied to conduct the meta-analysis. We will use Chi-squared and I^2^ tests to evaluate the heterogeneity of all studies included. I^2^ values >50 means high heterogeneity among studies included. If there is a high heterogeneity, we will conduct subgroup analyses to explore the possible causes

### 4.7 Assessment of reporting bias

If there are more than 10 included trials in this review, funnel plot will be used to discuss the reporting biases or small-study effects according Egger methods.

### 4.8 Data synthesis

We will use RevMan 5.3 software (The Cochrane Collaboration, Oxford, England) to calculate for data synthesis. If there no obvious statistical heterogeneity among the trails included, we will apply fixed effects model to perform in the analysis. However, the random effects model will be used, when apparent clinical heterogeneity among the trails included. Meanwhile, subgroup or sensitivity analysis will be conducted. α = 0.05 will be deemed statistically significant.

### 4.9 Subgroup analysis

Subgroup analysis will be conducted according to sex, smoking status, locations, histologic diagnosis, TNM stage, duration of TCM therapies, timing of TCM therapies, and chemotherapy regimens.

### 4.10 Sensitivity analysis

Sensitivity analysis will be conducted to explore the quality of studies of the document following sample size, the outcome of missing data, and methodological quality.

### 4.11 Ethics and dissemination

Ethical approval is not required because individual patient information will be not used. The authors will disseminate this systematic review through conference presentations and peer-review publications

## 5 Discussion

Although, many studies were conducted to show TCM therapies combined with chemotherapy associated with better DFS, OS, and quality of life,^[12–15]^ evidence still remains insufficient to demonstrate such combination of therapeutic method can cause better DFS and life quality as well as safety for patients with stage II to IIIA NSCLC. A systematic review which can provide the newest data should be conducted to show such combination of therapeutic method associated with better DFS, OS, and quality of life. Therefore, this protocol for a systematic review has to be displayed. We hope that our works will help clinicians with more convincing evidence about dealing with patients with postoperative II to IIIA NSCLC.

## Author contributions

Shuntai Chen and Runzhi Qi contributed to the conception of the study. Shuntai Chen, Xiwen Zhang and Zhenhua Zhang wrote the draft of manuscript, and was revised by Xing Zhang, Yupeng Xi and Honggang Zheng. The search strategy was developed by all of the authors. Shuntai Chen, Rui Liu and Qiujun Guo will search, extract data, assess the risk of bias, and complete the data synthesis. Baojin Hua will arbitrate in case of disagreement and ensure the absence of errors. All authors approved the publication of the protocol.

**Conceptualization:** Shuntai Chen, Runzhi Qi.

**Data curation:** Shuntai Chen, Rui Liu, Qiujun Guo.

**Formal analysis:** Shuntai Chen, Rui Liu, Qiujun Guo.

**Investigation:** Rui Liu, Qiujun Guo.

**Methodology:** Juling Jiang.

**Project administration:** Baojin Hua.

**Writing – original draft:** Shuntai Chen, Zhenhua Zhang, Xiwen Zhang.

**Writing – review & editing:** Xing Zhang, Yupeng Xi, Honggang Zheng.

Shuntai Chen orcid: 0000-0002-6927-7492.
